# Ordering of Hollow Ag-Au Nanospheres with Butterfly Wings as a Bio-template

**DOI:** 10.1038/s41598-018-27679-5

**Published:** 2018-06-18

**Authors:** Yu Guan, Huilan Su, Chengzhi Yang, Lingling Wu, Shikun Chen, Jiajun Gu, Wang Zhang, Di Zhang

**Affiliations:** State Key Lab of Metal Matrix Composites, School of Materials Science and Engineering, Shanghai Jiao Tong University, Shanghai, 200240 P. R. China

## Abstract

A biological template strategy is implemented for the fabrication of hollow noble metal composite nanospheres within the ordered array nanostructures by introducing butterfly wings to some convenient technique procedure. Butterfly wings are activated by ethylenediamine to increase the reactive sites on the chitin component, on which Ag nanoparticles are *in situ* formed and serve as “seeds” to direct further incorporation during the following impregnation procedure. Butterfly wings could function as bio-substrate to provide an ordered array and regulate the synthesis process by providing active reaction sites (e.g. -CONH- and -OH). Thus, hollow Ag-Au nanospheres are loaded on the wings’ surface layer and inside the ordered array nanostructures homogeneously, which would have potential applications in surface enhanced Raman scattering (SERS).

## Introduction

Noble metal materials with hollow structure have aroused much attention due to their promising applications in drug delivery^1^, sensors^2^, solar cells^3^ and nano-catalysis^4^. Hollow nanostructures usually exhibit better optical electronic^5–7^, magnetic^8,9^ and catalytic properties^10,11^ owing to larger specific surface, low density and small coefficients of thermal expansion. More efforts have been devoted to obtain hollow nanostructures of noble metal. S. Guo *et al*.^12^ prepared hollow Pt nanosphere and hollow Au/Pt nanostructures using Ag nanoparticles as templates. K.G. Gopchandran *et al*.^13^ synthesized Au-Ag hollow nanostructures by galvanic replacement mechanism. Y. Xia and coworkers^14^ prepared Au nanocages using Ag nanocubes as sacrificial templates. Recently, it is reported that the properties of noble nanoparticles can be further enhanced when arranged in an orderly structure. K. Kneipp *et al*.^15^ found that the SERS enhancement factor of arranged gold nanoparticles could reach 10^14^ in contrast to the original 10^3^ produced by single particle. Hence hollow noble metal with ordered array should have greater potential in applications like surface-enhanced Raman scattering spectra^16^, biosensors^17^, solar cells^18^ and nonlinear optics^19^.

A bio-templated strategy may be a potential way to render nanostructures an ordered array. Some biological tissues such as exocarp^20^, pollen and Lepidopteran wings^21–23^ present extremely fine hierarchical structures after hundreds of millions of years of evolution. Considerable efforts have been done to synthesize ordered nanostructures by replicating the structure of bio-template. F. C. Meldrum^24^ prepared porous gold structures with nearly regular 15 μm channels by utilizing sea urchins as templates. Y. Tan^22^ and coworkers obtained seven single metals with hierarchical sub-micrometer structures by mimicking butterfly wing scales and proved good property of SERS of the products. However, it is rare to regulate composition and structure of the composition unit of these ordered array nanostructures.

Herein, we introduce a facile synthesis strategy to control the composition unit as hollow Ag-Au nanospheres with ordered array under regulation of butterfly wings. The butterfly wing scales could function as bio-substrate upon which hollow Ag-Au nanospheres could anchor to form an ordered array structures. The details of hollow Ag-Au nanospheres and function of butterfly wings were investigated through some technology. This work may bring new opportunities for the design and fabrication of nanostructures with ordered array possessing interesting physicochemical properties.

## Results and Discussion

**Figure 1:**
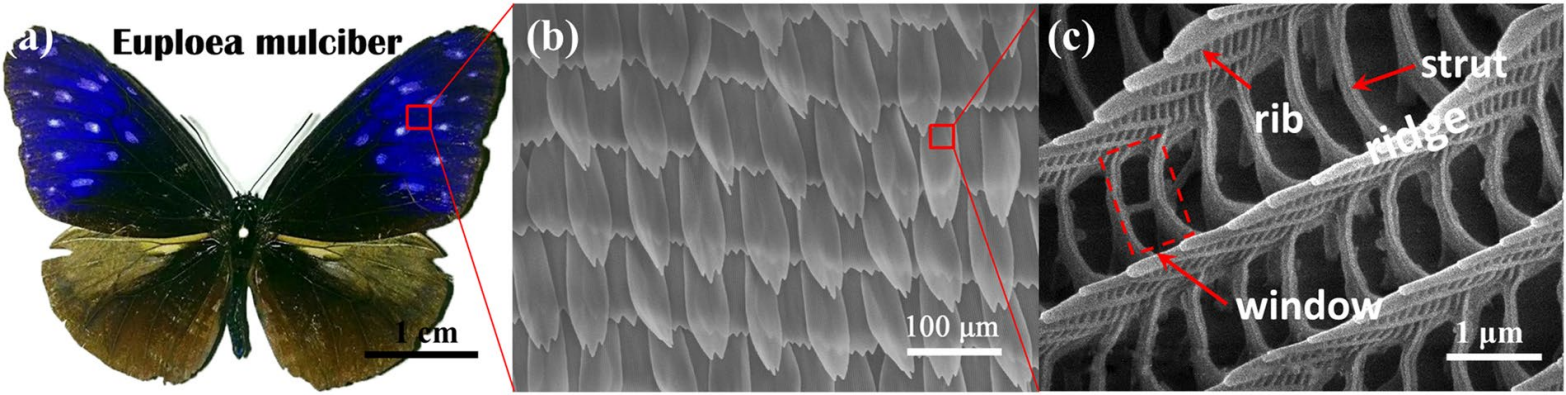
Morphology and microstructure of Euploea mulciber (**a**) digital photo of whole butterfly (**b**,**c**) SEM images of original wing scale.

Forewings (Fig. 1(a)) of Euploea mulciber provide bio-template of nanostructures in an orderly arrangement. The forewings exhibit blue-green iridescence only at parts where the incident lights reflected on the surfaces, owing to their special 3D periodic sub-microstructures^25^. These 3D sub-microstructures including ridges, ribs and struts, are further divided up to numerous periodic window structures by horizontal and vertical struts between the parallel-aligned ridges. The main components of wings include chitin and a small amount of protein, which can provide some reaction hotspots^26^.

**Figure 2:**
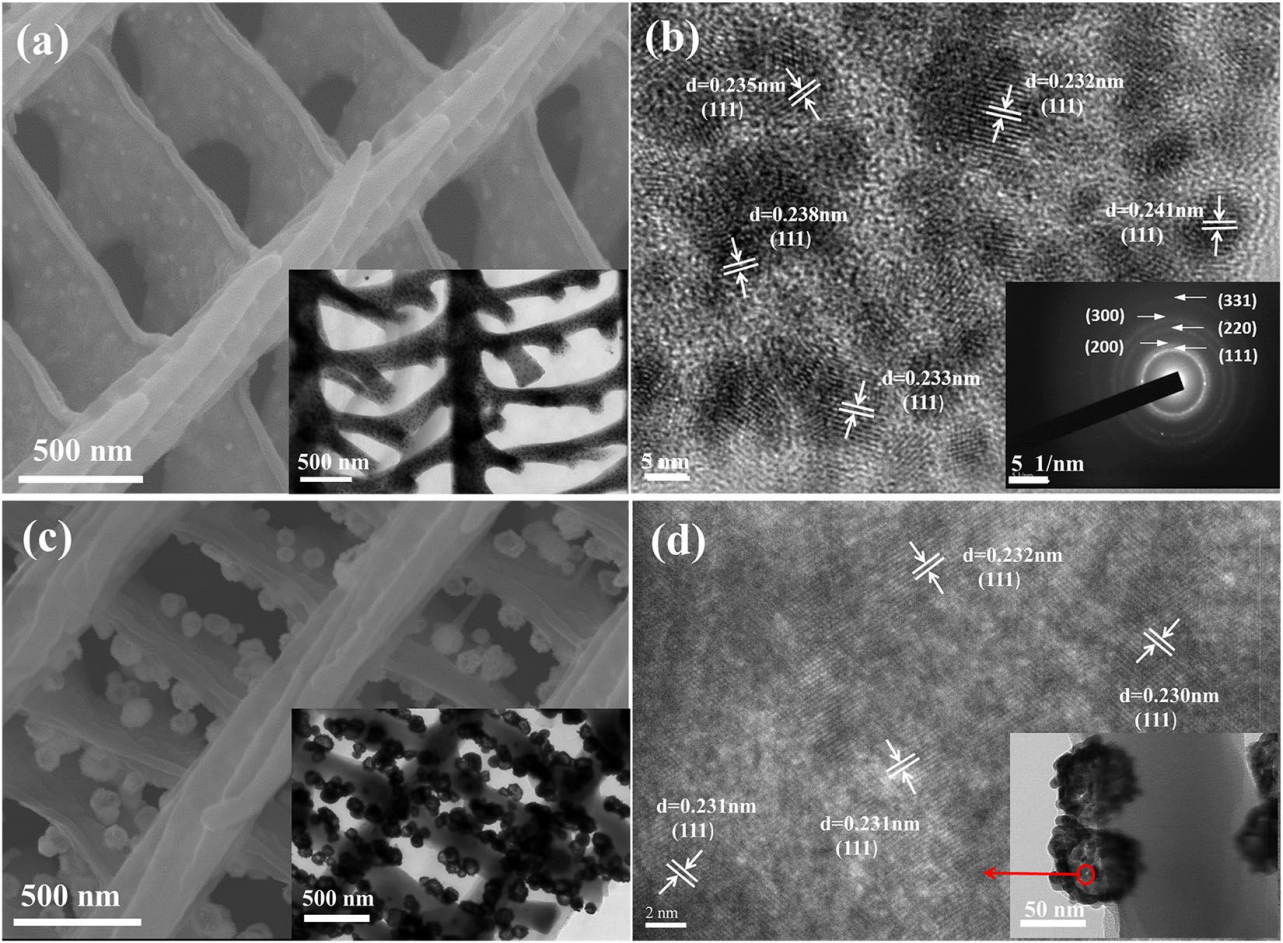
(**a**) SEM image of Ag/wing materials, inset is TEM image of Ag/wing materials (**b**) HRTEM image of Ag/wing materials, inset is SAED patterns of the incorporated nano-Ag (**c**) SEM images of Ag-Au/wing materials, inset is TEM image of Ag-Au/wing materials (**d**) HRTEM image of a single hollow Ag-Au sphere.

Figure 2(a) shows the SEM images of intermediate product: Ag/wing. As can be seen, the intermediate product is covered with a layer of particles distributing on the “ridge” and “strut” uniformly, rougher than that of the original wings in Fig. 1. The mapping image of the intermediate product can be find in Figure S2 in supplementary information. As revealed by the HRTEM images in Fig. 2(b), the particles are nearly round with diameter 5–10 nm. The corresponding SAED patterns (Fig. 2(b) inset image) match well with the face-centered cubic (fcc) Ag structure reported in JCPDS cards 040783, and the relevant planes are indexed as (1 1 1), (2 0 0), (2 2 0), (3 0 0), and (3 1 1). Besides, HRTEM image in Fig. 2(b) exhibits clear lattice fringes with *d* spacing 0.23 nm, corresponding to the (1 1 1) plane of fcc Ag structure, which indicates that (111) plane is the most stable crystal plane. The less dark areas among the nanoparticles are wing scales. The structure of wing scale keeps intact and nano-Ag are embedded in wing scales matrix. Hollow Ag-Au nanospheres (Fig. 2(c)) on the wing scales are prepared based on the wing-Ag product. The distribution of hollow Ag-Au nanosphere on butterfly wings is investigated by FESEM and TEM observation. It can be seen that about 60 nm nanospheres distribute on the “ridge” and “strut” uniformly. Figure 2(d) is HRTEM image of a part of a single hollow Ag-Au sphere, it cannot be confirmed whether they are Au or Ag in spite of the 0.23 nm of *d* spacing. Overall, about 60 nm hollow nanospheres with the ordered array nanostructures are synthesized with 5–10 nm fcc Ag *in-situ* grown on the surface of wings as “seeds”.

**Figure 3:**
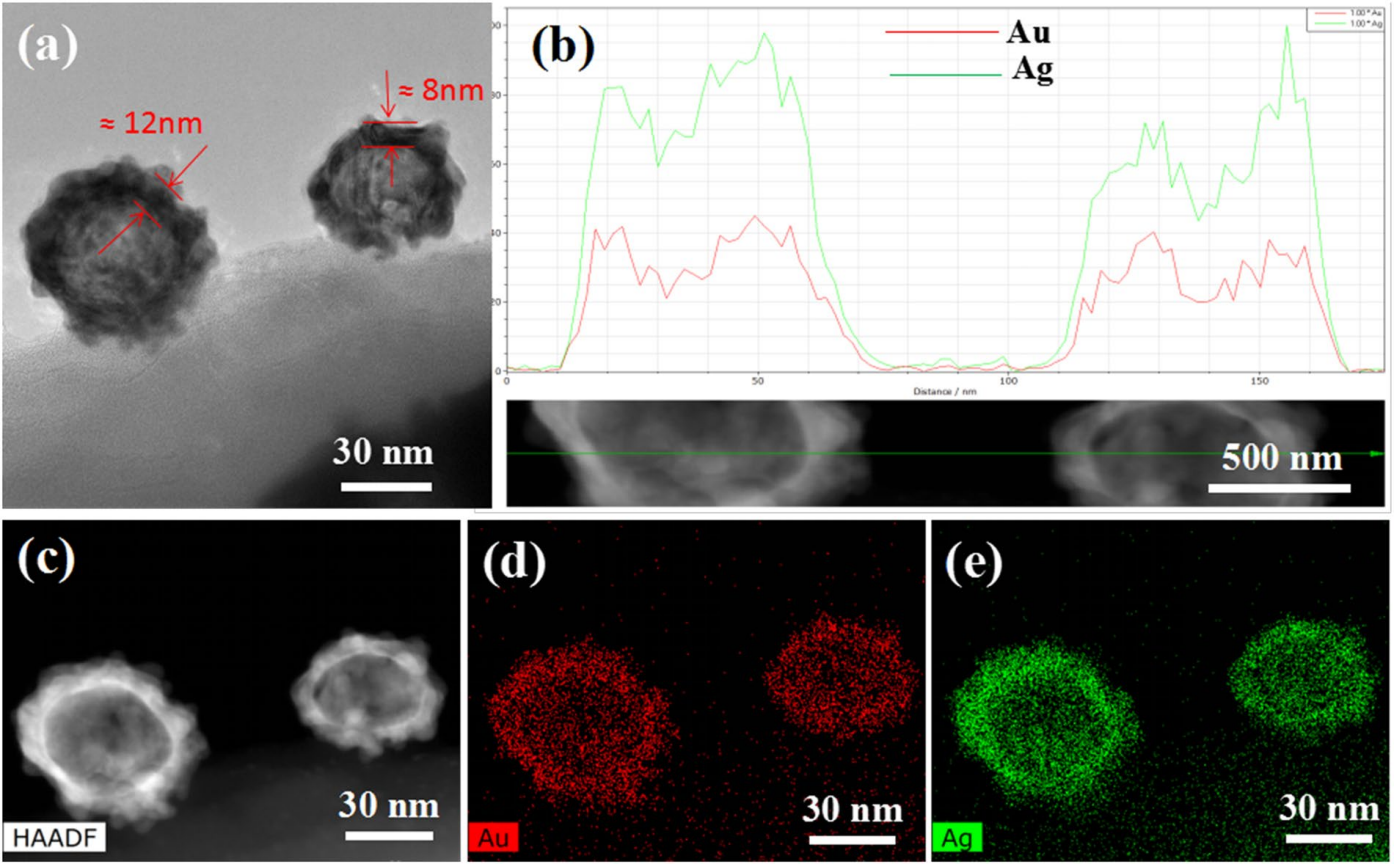
Morphology and composition of Ag-Au nanosphere on wing scale. (**a**) STEM images (**b**) result of line scanning (**c**) STEM-EDX elemental mapping image of (**d**) Au and (**e**) Ag.

Figure 3(a) presents a typical hollow Ag-Au nanosphere incorporated into butterfly wings structure. Line scanning (Fig. 3(b)) exhibits a board peak of Au and Ag in the center and two intensive peaks on both sides, further confirming that Au and Ag tend to be distributed at the periphery and form a hollow structure internally. TEM images in Fig. 3(a) show that the thickness of Ag-Au shell is about 10 nm. Atomic percentage of Au and Ag are 44.2% and 55.8%, respectively, according to the semi-quantitatively analysis. The concentration of HAuCl_4_ is about 0.2 wt% and one can speculate that the thickness and element content of hollow Ag-Au nanospheres could be regulated by the concentration of HAuCl_4_ solution, as can be seen in Figure S3 in supplementary information. Elemental analyses of hollow Ag-Au nanospheres were performed by energy disperse X-ray analysis (EDX) technique. The mapping images (Fig. 3(c,d,e)) confirm the presence of elemental Ag and Au in the nanocomposite. Besides, both Ag and Au distribute nearly homogeneously across the nanoparticles.

**Figure 4:**
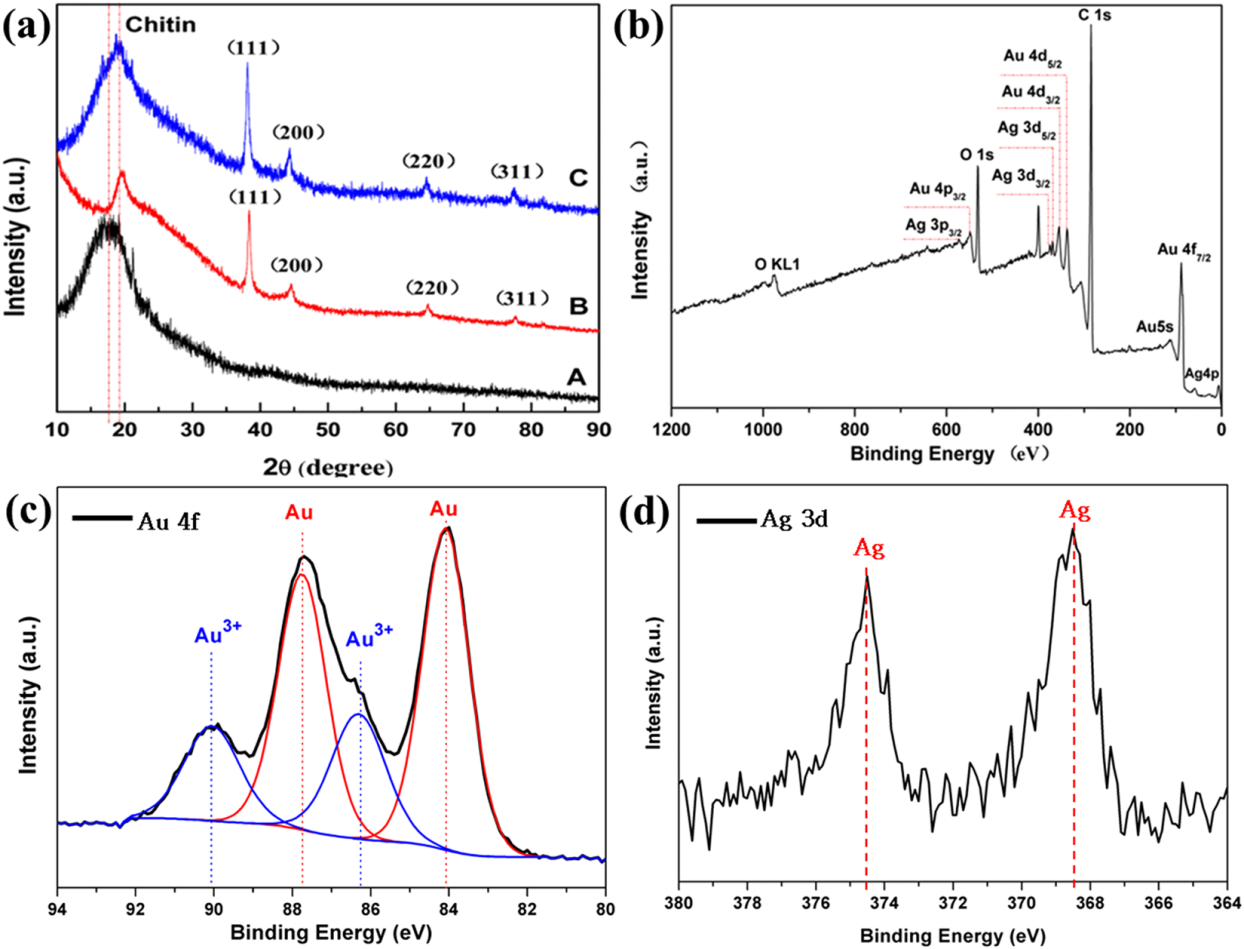
(**a**) XRD patterns of as-prepared products. A: original butterfly wing, B: Ag/wings, C:Au-Ag/wings (**b**) XPS patterns of final product (**c**) XPS patterns of Au 4 f (**d**) XPS patterns of Ag 3d.

Figure 4(a) shows the XRD patterns of the Ag-Au/wing product compared with Ag/wing and original butterfly wing. The result shows that the natural butterfly wing and the processed butterfly wing exhibit the diffractive peaks of crystalline domains around 20°. It indicates that the samples have the same components of chitin, and chitin doesn’t change in the process. In addition, the diffraction pattern of Ag/wing has four peaks at 38.1°, 44.2°, 64.4° and 77.5°, corresponding to (1 1 1), (2 0 0), (2 2 0), and (3 1 1) of face-centered cubic (fcc) structure of Ag (JCPDS file No. 04-0783), respectively. The intensity of (1 1 1) plane is twice the value from standard card (JCPDS file No. 04-0783), which proved that (1 1 1) crystal face is the most stable crystal face of nano-Ag seeds. Line C represents the hollow Ag-Au nanospheres with ordered array nanostructures, the peak intensity increases and the peak position moves to the left slightly compared with Line B, which indicates the formation of Ag-Au nanocomposites. To confirm these results, XPS analyses (Fig. 4(b)) were performed on the Ag-Au/wings systems. Figure 4(c) shows the XPS Au 4 f spectrum of the Ag-Au/wings systems. The result suggests that Au and Au^3+^ coexist in the final product, and the relative contents of Au and Au^3+^ are 69.3% and 30.7% respectively. The binding energy of the Au are at 84.1 eV and 87.8 eV, and the splitting between the 4f_7/2_ and 4f_5/2_ lines is equal to 3.7 eV, corresponding to the binding energy of standard bulk Au0 (84.0 eV and 87.7 eV)^27^. Such shift to higher energy is commonly observed for metallic nanoparticles and is attributed to geometry and/or confinement effects^28^. The XPS Ag 3d spectrum is shown in Fig. 4(d). The Ag 3d spectrum exhibits two contributions, between the 3d_5/2_ (374.5 eV) and 3d_3/2_ (368.5 eV) lines is equal to 6.0 eV, corresponding to Ag0 electronic state 374.2 eV and 368.2 eV reported in the literature^29^. Overall, the XPS analyses confirm the Ag0 valence state, Au0 and Au^3+^ of the Ag-Au nanospheres.

**Figure 5:**
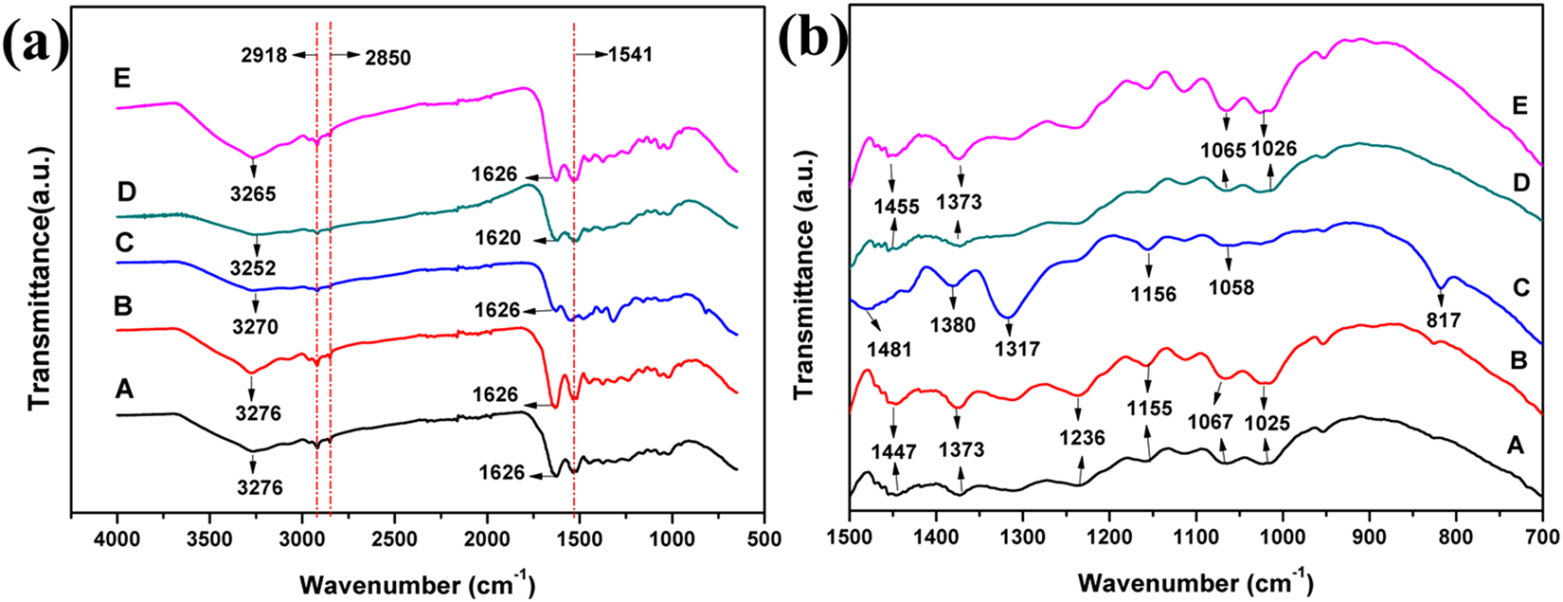
ATR-FTIR spectra of (**a**) the original chitin-based butterfly wings (**b**) butterfly wings washed by HNO_3_ (**c**) the EDA activated wings (**d**) the Ag/wings (**e**) the Ag-Au/wings3.

FTIR spectra were performed to investigate the mechanism of synthesis of Ag-Au nanoparticles and the participation role of butterfly wing template. ATR-FTIR spectra (Fig. 5(a)) of the original butterfly wings show peaks around 1626 cm^−1^ and 1541 cm^−1^, assigned to the amide I band of chitin due to C=O bending vibration from -CONH- and the amide II band of chitin due to stretching vibration of C-N and N-H^30^. The peaks around 1067 cm^−1^, 1155 cm^−1^, 1541 cm^−1^ can be assigned to the vibration characteristics of C-O of chitin. Absorption band between 3250–3450 cm^−1^ is vibration characteristics of N-H and stretching vibration of O-H. Hence, there have been some active sites (such as -CONH- and -OH) on the protein/chitin components of the original butterfly wing already^26^. Comparing curves of A and B, the main peaks have no change, indicating that nitric acid only plays the role of washing. From the change between curve B and curve C, it is clear that after the wings are activated by EDA, the peak around 3276 cm^−1^ moves to 3267 cm^−1^ and the peak at 1457 cm^−1^ shifts to 1481 cm^−1^ with increased absorption. Moreover, the peak at 817 cm^−1^ (N-H vibration), and the peak at 1317 cm^−1^ (N-H vibration) are found on curve C, indicating there are more active sites due to activation of ethylenediamine. After the formation of nano-Ag on wing scales, the peak around 1625 cm^−1^ of C=O bending vibration from -CONH- shifts to 1629 cm^−1^, and the peak at 1481 cm^−1^ of -OH stretching vibration from free -COOH moved to 1455 cm^−1^, suggesting there might be some coordination reactions between [Ag (NH_3_)_2_]^+^ and carboxyl groups and the amide I. The peak at 1058 cm^−1^ of -OH stretching vibration shifts to a wider peak at 1069 cm^−1^, which further indicates the possible existence of coordination reaction between -NH_2_ and Ag ions. Butterfly wings acts as a substrate by binding [Ag (NH_3_)_2_]^+^ with coordination reaction sites -CONH- and -OH.

**Figure 6:**
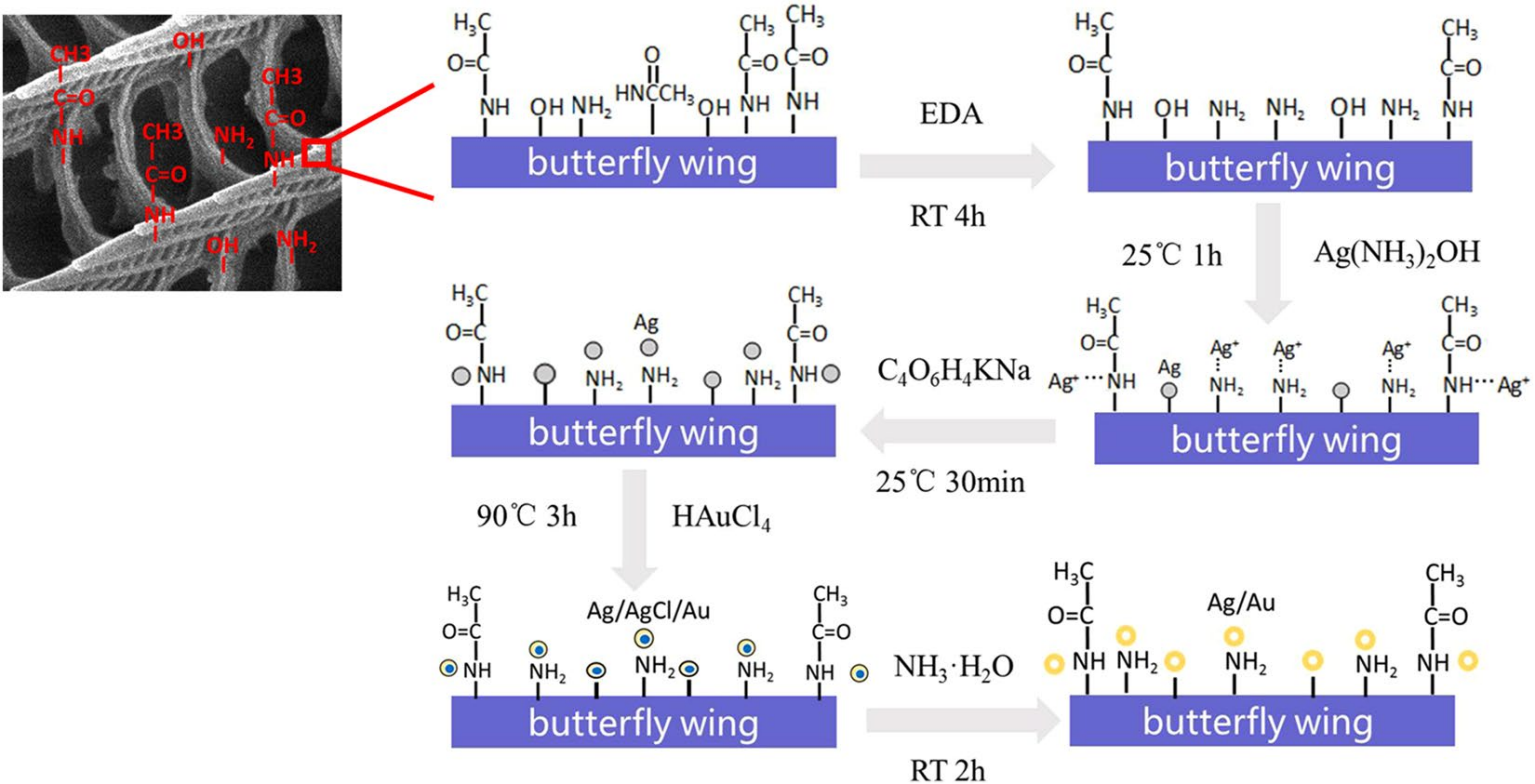
Mechanism of butterfly wings synthesis and modification of hollow Au-Ag nanospheres.

According to the ATR-FTIR spectra results, we figure out the schematic procedures for butterfly wings to regulate the fabrication of hollow Ag-Au nano-spheres (Fig. 6). The original butterfly wings include active functional groups (such as -CONH- and -OH). After activation with ethylenediamine, more functional groups (-NH_2_, -OH) generate on the butterfly wing. When activated butterfly wings are immersed in silver-ammonia solution, some [Ag(NH_3_)_2_]^+^ are trapped on wings by chelation with -OH, -NH_2_ and -CONH- sites provided by chitin and other small nano-Ag are formed *in-situ* on the surface of wings. Here wings act as both the template and supporting substrate. Then [Ag (NH_3_)_2_]^+^ trapped on wings are reduced to nano-Ag by reducing agent (Potassium tartrate). The prepared Ag nanoparticles can serve as seeds of synthesis of Au-Ag nanoparticle. During the immersion of Ag/wing in HAuCl_4_ solution, Au III is reduced to nano-Au by nano-Ag because the AuCl_4_^-^/Au (0.99 V vs NHE) is more positive than that of AgCl/Ag (0.22 V vs NHE).

**Figure 7:**
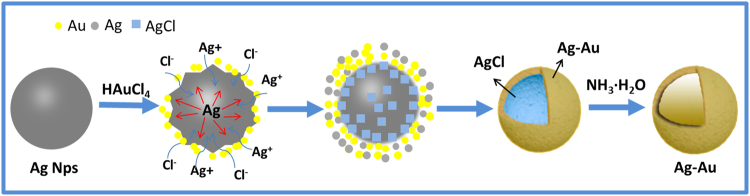
The formation schematic of hollow Au-Ag nanospheres.

We hypothesize the mechanism shown in Fig. 7 for hollow Au-Ag nanospheres’ formation. The produced Au and Ag are distributed to the surface of nanosphere and interior is AgCl when immersing the Ag-wings sample into HAuCl_4_ solution. Hollow Au-Ag nanosphere within the ordered array nanostructures are produced after AgCl was further dissolved. We begin by describing the mechanism for nanosphere’s formation. When Ag nanoparticles with (1 1 1) crystal faces (Fig. 2) react with a small amount of HAuCl_4_ solution, Ag dissolution occurs at all cube corners for Ag nanoparticles^14^ since the (1 1 1) corners are more stable in comparison to the other faces. As the reaction proceeds, Ag is oxidized and electrons are stripped, defects are introduced into the structure due to reaction stoichiometry: three Ag atoms are removed with deposition of one Au atom (3Ag + HAuCl_4_ → 3AgCl + Au + HCl)^31^. The released electrons migrate to the nanoparticles faces and are captured by AuCl_4_^-^, generating Au atoms that grow on the nanoparticles epitaxially and silver chloride precipitates come into being at the same time. Thus, Ag/Au/AgCl nanoparticles are produced given the mutual solubility of Cl, Ag and Au and the high diffusion rates at the reaction temperature. Finally, the AgCl precipitate is removed and hollow Ag-Au nanospheres remained incorporating into the ordered array nanostructures within butterfly wing.

**Figure 8:**
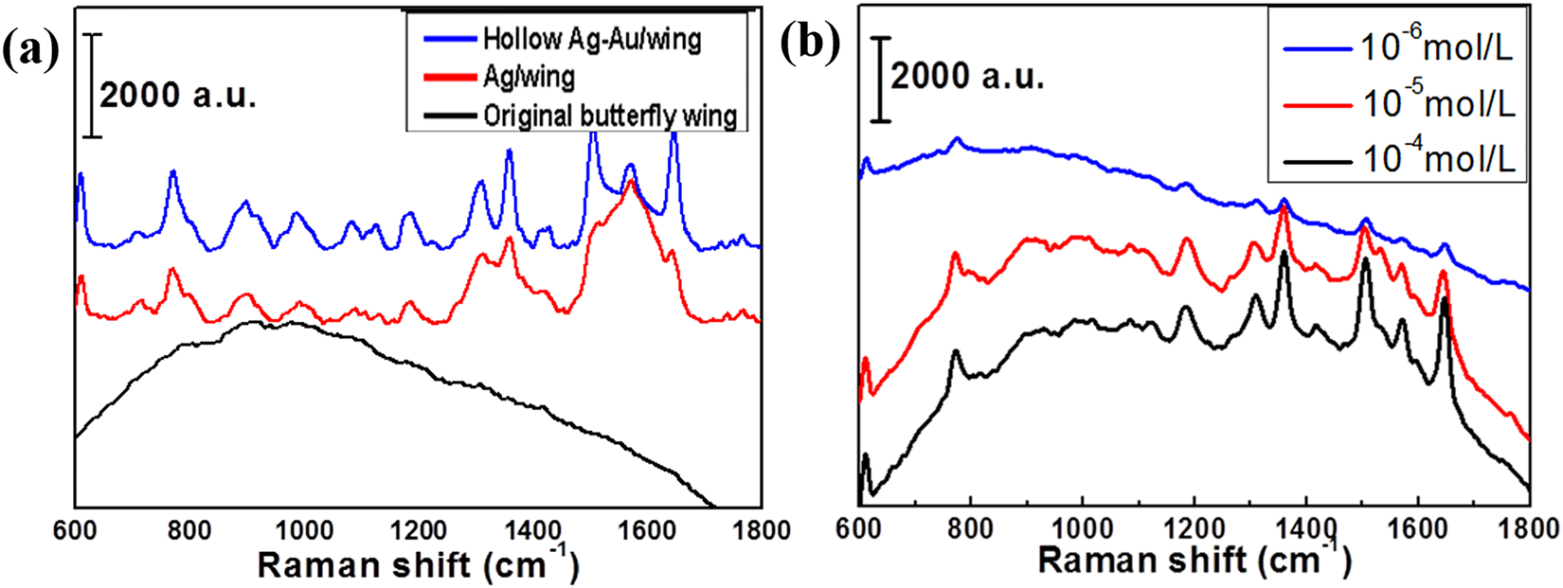
(**a**) Comparison of Raman signal from R6G on original butterfly wing, Ag/wing and hollow Ag-Au/wing, R6G concentration: 10^−3^ mol/L; (**b**) Comparison of Raman signals from different concentrations of R6G on hollow Ag-Au/wing, R6G concentrations: 10^−4^ mol/L, 10^−5^ mol/L, 10^−6^ mol/L.

The SERS property of the Ag-Au/wing material was tested at 532 nm wavelength by Senterra R200-L dispersive laser confocal Raman spectrometer using Rhodamine 6 G (R6G) as a Raman signal molecule. We judge the Raman properties by the intensity of the signal peak. Figure 8(a) is comparison of Raman signal from R6G at concentration of 10^−3^ mol/L on original butterfly wing, Ag/wing, Ag-Au/wing. One can see that the corresponding curve of the original butterfly wing is relatively smooth, which indicates that the original butterfly wing has no enhancement of the Raman signal from R6G. Ag/wing and hollow Ag-Au/wing can detect R6G signal, and the signal of hollow Ag-Au/wing is slightly stronger. Figure 8(b) shows that the hollow Ag-Ag/wing substrate can detect the Raman signal from R6G at concentration of 10^−6^ mol/L successfully, which is 10^2^ times higher than that of Ag/wing. The limit of detection for R6G is calculated to be 8.67 × 10^−10^ mol/L using the method reported by C. Zhu *et al*.^32^ and Figure S5 in supplementary information. Here, the advanced SERS performance of hollow Ag-Au/wings probably results from the following factors. First, hollow Ag-Au particles generate more hotspots, resulting in an enhanced SERS effect^13^. Second, the hotspots formed by the combination of Ag-Au nanoparticles with the micro-nanostructure of the butterfly wing also contribute to the improvement of SERS properties. Therefore, the materials with hollow structures and micro-nano structures may have great potential as new SERS substrates.

## Conclusions

A facile synthesis strategy is used to fabricate Ag-Au composite with ordered array nanostructures within butterfly wings and control the constituent units to be hollow Au-Ag nanospheres. Butterfly wing scales function as bio-template to stabilize the nanocrystal and provide active functional groups (-CONH- and -OH) on which nanoparticles can *in situ* grow. The decomposition and reduction route provides a new method for synthesis of hollow metallic spheres. Hollow Ag-Au nanospheres with ordered array nanostructures are produced by the diffusion of Cl, Ag and Au with participation of butterfly wings, which would have significant potential applications such as SERS substrates. The synthetic technique and mechanistic understanding may provide new opportunities to design and fabricate heterogeneous nanostructures with interesting physicochemical properties.

## Experimental

In a typical process, butterfly wings were first immersed in diluted nitric acid (13 vol. %) for 1 h and then in ethylenediamine for 4 h (amination) to remove mineralized impurities and activate. Immerse the aminated samples in an aqueous solution of [Ag(NH_3_)_2_] OH (0.03 mol/L) for 1 h, then expose in 50 g/L aqueous solution of potassium sodium tartrate for 30 min. Ag-NP seeds were formed on the aminated bio-surface. Immerse the Ag-NP-functionalized butterfly wings into HAuCl4 (0.2 wt%) solutions at 90 °C. Immerse product into ammonia for 2 h at room temperature, rinse them with deionized water, and dry in air. The whole process should be in the dark to avoid the influence of the light. All chemicals were of analytical grade from the Sinopharm Chemical Reagent Co., Ltd. and were used as starting materials without further purification.

X-ray diffraction (XRD) patterns were carried out on Ultima IV instrument operating at a voltage of 40 kV and a current of 30 mA. X-ray photoelectron spectroscopy (XPS) studies were recorded on AXIS Ultra DLD spectrometer using standard Al Kα X-ray source (150 W) and an analyser pass energy of 20 eV. Samples were mounted using double-sided adhesive tape and binding energies were referenced to the C 1 s peak at 284.8 eV of the surface adventitious carbon. FESEM images were recorded on S-4800 field emission scanning electron microscope operated at an acceleration voltage of 10 kV. FETEM were performed on a JEOL JEM-2100F instrument operated at an acceleration voltage of 200 kV. FT-IR spectra were obtained by NICOLET 6700. The Raman signal of the sample was measured by a dispersive laser confocal Raman spectrometer Senterra R200-L.

## Electronic supplementary material

Supplementary Information
