# Supplementary material for: Ordering of Hollow Ag-Au Nanospheres with Butterfly Wings as a Bio-template
Source: Sci Rep. 2018 Jun 18;8:9261. doi: 10.1038/s41598-018-27679-5 (PMC6006350; doi:10.1038/s41598-018-27679-5)
Supplement: Supplementary file 1 — Supplementary Information [file 41598_2018_27679_MOESM1_ESM.pdf]

Supporting Information Available

Ordering of Hollow Ag-Au Nanospheres with Butterfly Wings as a Bio-template

Yu Guan, Huilan Su*, Chengzhi Yang, Lingling Wu, Shikun Chen, Jiajun Gu, Wang Zhang, Di Zhang*

State Key Lab of Metal Matrix Composites, School of Materials Science and Engineering, Shanghai Jiao Tong University, Shanghai 200240, P. R. China

Fax：+8634202749　Tel：+862154745872

E-mail: hlsu@sjtu.edu.cn, zhangdi@sjtu.edu.cn

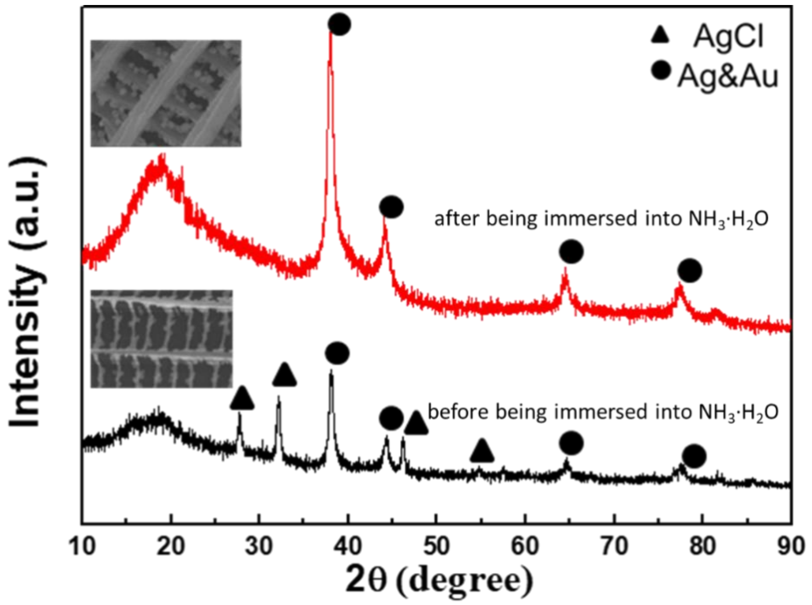

Figure S1. XRD patterns of Ag-Au/wings before being immersed into $NH_3 \cdot H_2O$ and after being immersed into $NH_3 \cdot H_2O$.

Figure S1 shows the XRD patterns of the Ag-Au/wings product XRD patterns of Ag-Au/wings before being immersed into $NH_3 \cdot H_2O$ and after being immersed into $NH_3 \cdot H_2O$. The result shows that the prepared wings before being immersed into $NH_3 \cdot H_2O$ exhibit the diffractive peaks at 27.8°, 32.2°, 38.1°, 44.2°, 46.2°, 54.8°, 64.4° and 77.5°. The diffraction patterns of wings at 27.8°, 32.2°, 46.2° and 54.8° are corresponding to (1 1 1), (2 0 0), (2 2 0), and (3 1 1) of AgCl (JCPDS file No. 31-1238), respectively. The diffraction patterns at 38.1°, 44.2°, 64.4° and 77.5° are corresponding to (1 1 1), (2 0 0), (2 2 0), and (3 1 1) of face-centered cubic (fcc) structure of Ag (JCPDS file No. 04-0783), respectively. After being immersed into $NH_3 \cdot H_2O$, there are only the diffractive peaks at 38.1°, 44.2°, 64.4° and 77.5°, which corresponding to (1 1 1), (2 0 0), (2 2 0), and (3 1 1) of face-centered cubic (fcc) structure of Ag (JCPDS file No. 04-0783). The results prove that $NH_3 \cdot H_2O$ could dissolve AgCl to further form hollow Ag-Au nanoparticles.

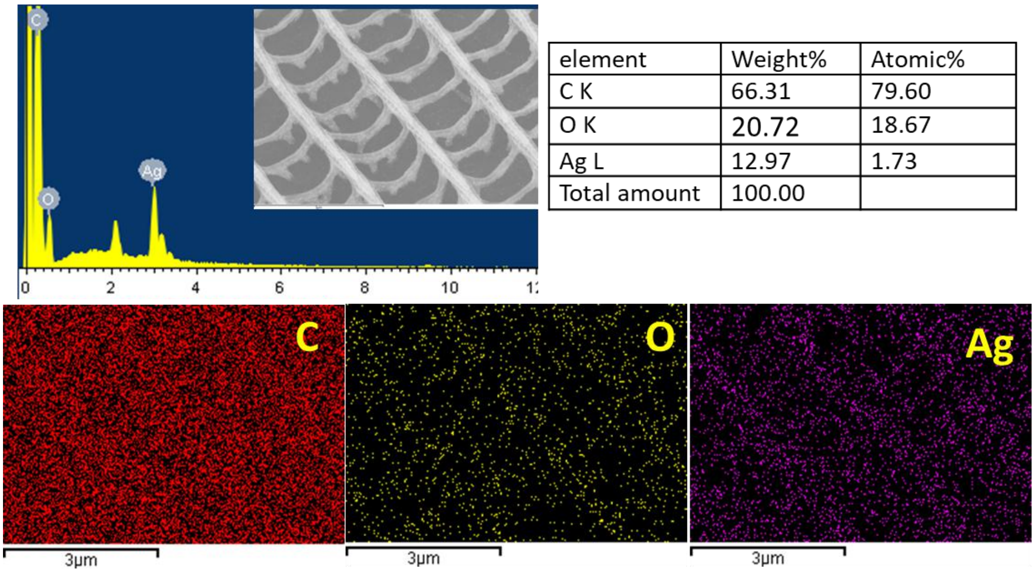

element	Weight%	Atomic%
C K	66.31	79.60
O K	20.72	18.67
Ag L	12.97	1.73
Total amount	100.00	

Figure S2. Mapping image of Ag/wings

The energy dispersive X-ray (EDX) spectra and mapping informations reveal that Ag/wings are mainly composed of C, O and Ag, in which C and O are the components of chitin from the butterfly wing. The distribution of Ag element shows the overall characteristics of the butterfly wing structure.

The formation process of nanostructured Ag-Au can be expressed in the following equations. Equations (1) and (2) are the formation process of [Ag(NH3)2]$^{+}$OH solution. [Ag(NH3)2]$^{+}$OH solution is reduced through Equations (3) .As the reaction equations (4) proceeding, Ag is oxidized and electrons are stripped and forming Ag-Au-AgCl particles, defects are introduced into the structure due to reaction stoichiometry. Finally, the AgCl precipitate was removed according equation (5) and remained hollow Ag-Au nanospheres incorporating into the ordered array nanostructures within butterfly.

$$AgNO_3+NH_3\cdot H_2O=AgOH\downarrow+NH_4NO_3 \quad (1)$$

$$AgOH+2NH_3\cdot H_2O = [Ag(NH_3)_2]^+ + 2H_2O+OH^- \quad (2)$$

$$[Ag(NH_3)_2]^+ + Reduction=Ag \quad (3)$$

$$3Ag + HAuCl_4 \rightarrow 3AgCl + Au + HCl \quad (4)$$

$$AgCl+ 2NH_3\cdot H_2O \rightarrow [Ag(NH_3)_2]^+ + Cl^- + 2H_2O \quad (5)$$

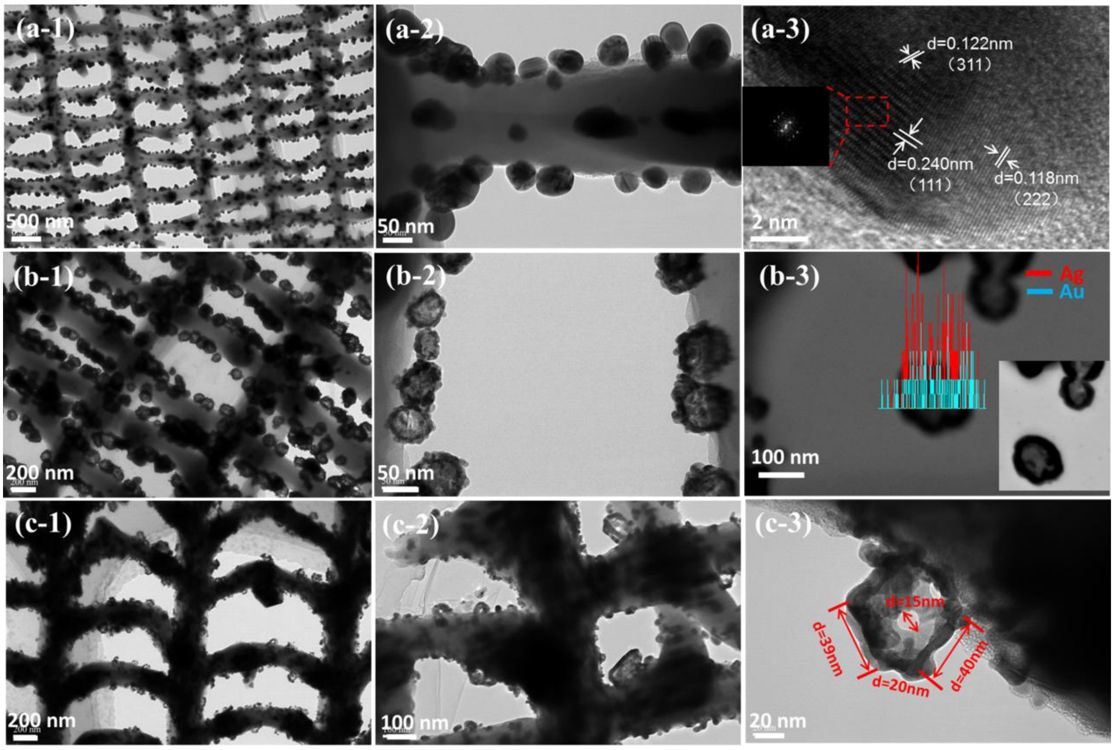

Figure S3. Morphology of Ag-Au/wing materials. (a-1) (a-2) (a-3) are TEM images and HRTEM image of nano Ag-Au/wing, (b-1) (b-2) (b-3) are TEM images and mapping results of hollow Ag-Au/wing, (c-1) (c-2) (c-3) are TEM images of irregular Ag-Au/wing

Various nanostructured Ag-Au with different morphologies and sizes, such as 30~50nm solid spherical, 50~80nm hollow spherical, irregular Ag-Au particles, can be prepared and loaded both on the wings' surface layer and inside the ordered array nanostructures homogeneously by changing the process parameters in the nature bio-template strategy (see figure S3).

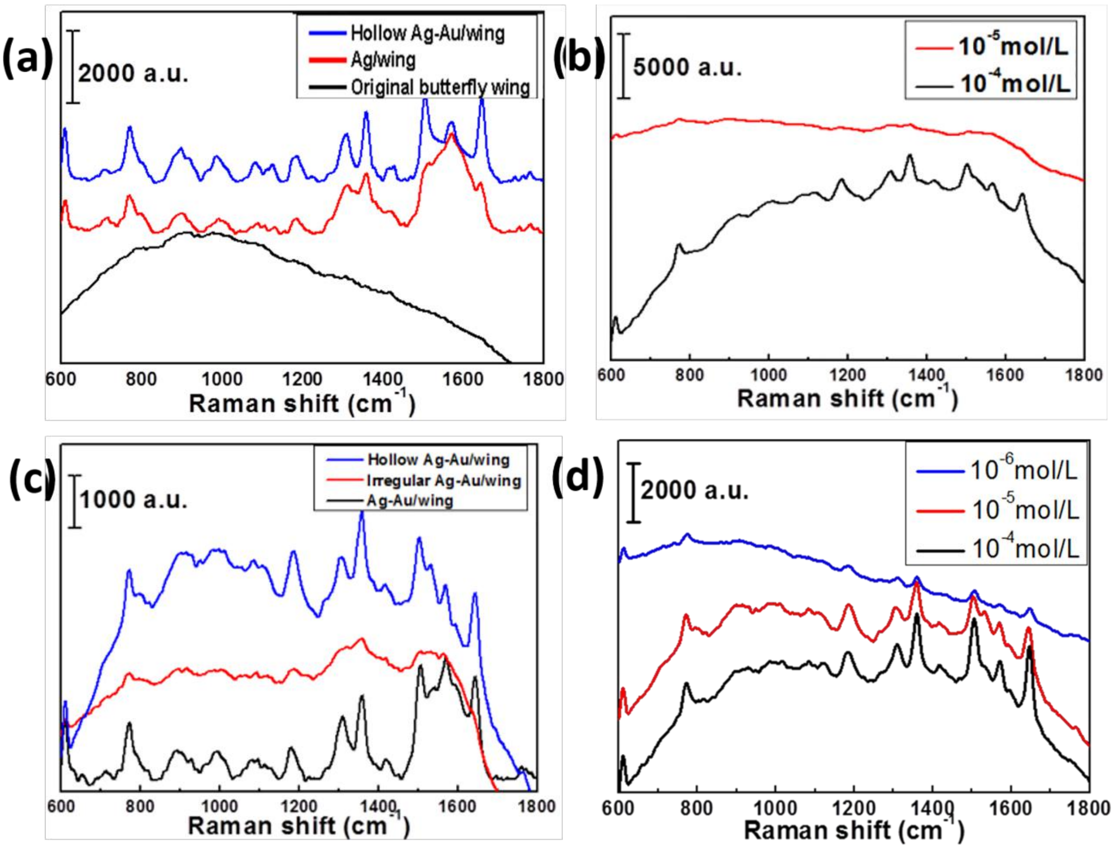

Figure S4. (a) Comparison of Raman signals from R6G on original butterfly wing, Ag/wing, Ag-Au/wing, R6G concentration: 10^{-3}mol/L. (b) Comparison of Raman signals from different concentrations of R6G on Ag/wing substrate, R6G concentrations: 10^{-4} mol / L, 10^{-5} mol/L. (c) Comparison of Raman signals from R6G on Ag-Au/wing substrates of three different morphologies, R6G concentration: 10^{-3} mol/L. (d) Comparison of Raman signals from different concentrations of R6G on hollow Ag-Ag/wing, R6G concentrations: 10^{-4} mol / L, 10^{-5} mol/L, 10^{-6} mol / L.

Figure S4(a) gives the comparison of Raman signal from R6G on original butterfly wing, Ag/wing, and hollow Ag-Au/wing. One can see that the corresponding curve of the original butterfly wing is relatively smooth which indicating that original butterfly wing has no any enhanced Raman signals of rhodamine 6G molecular. Ag/wing can detect minimum concentration of R6G signal is 10^{-4} (Figure S4(b)). Figure S4(c) offers Raman signals of the sample substrate embedded in the butterfly-wing structure with Ag-Au/wings, hollow Ag-Au/wings and irregular Ag-Au/wings. We can see that the hollow nanostructured Ag-Au shows the best SERS performance among the three different morphologies of Ag-Au/wings materials. Therefore, Raman enhancement effect of the hollow Ag-Au/wing matrix is better than the other two morphologies. The detection concentration of rhodamine 6G signal can

be achieved by 10^{-6} mol/L (see Figure S4(d)), which is better than the other two morphologies.

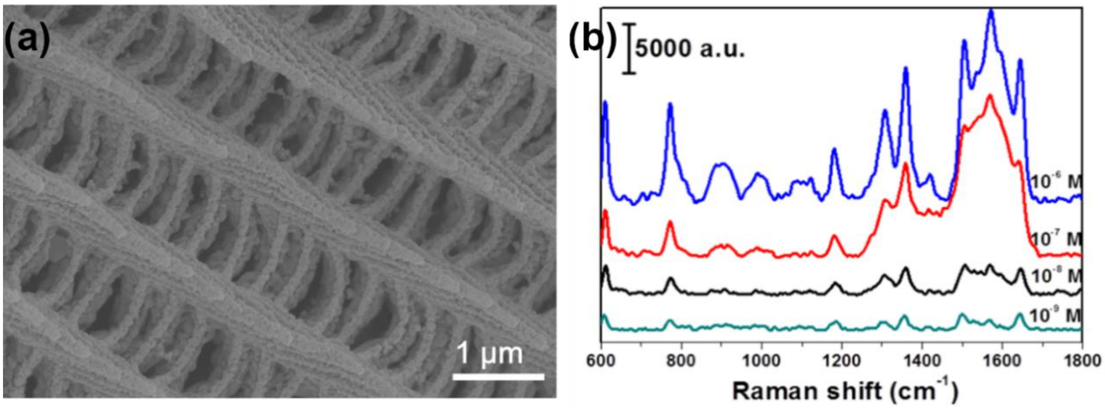

Figure S5. (a) SEM image of high-density samples of Ag-Au/wing. (b) SERS signals of R6G, R6G concentrations: 10^{-6} mol/L, 10^{-7} mol/L, 10^{-8} mol/L,10^{-9} mol/L

The density of Ag-Au nanoparticles can be controlled by changing process parameters and even process method, such as concentration of $HAuCl_4$ solution and impregnation order. We studied the SERS properties with Rhodamine 6G as the signaling molecule and used the strength of signal peak as the standard. We found that the density of Ag-Au nanoparticles will impact the SERS properties. Figure S5(b) is the comparison of SERS signals from R6G at different concentration, by which we can calculate the limit of detection for R6G of our sample is 8.67×10^{-10}mol/L.
